# Automatic Analysis of Ultrasound Images to Estimate Subcutaneous and Visceral Fat and Muscle Tissue in Patients with Suspected Malnutrition

**DOI:** 10.3390/diagnostics15080988

**Published:** 2025-04-13

**Authors:** Antonio Cuesta-Vargas, José María Arjona-Caballero, Gabriel Olveira, Daniel de Luis Román, Diego Bellido-Guerrero, Jose Manuel García-Almeida

**Affiliations:** 1 Clinimetria Research Group, Department of Physiotherapy, Faculty of Health Sciences, Universidad de Málaga, 29071 Malaga, Spain; jacuesta@uma.es; 2 Instituto de Investigación Biomédica de Málaga y Plataforma en Nanomedicina-IBIMA Plataforma BIONAND, 29590 Malaga, Spain; gabolvfus@uma.es (G.O.); jgarciaalmeida@gmail.com (J.M.G.-A.); 3 Department of Endocrinology and Nutrition, Hospital Regional Universitario de Málaga, 29010 Malaga, Spain; 4 Department of Medicine and Dermatology, Faculty of Medicine, Universidad de Malaga, 29010 Malaga, Spain; 5 Centro de Investigación Biomédica en Red (CIBER) de Diabetes y Enfermedades Metabólicas Asociadas, Instituto de Salud Carlos III, 29010 Málaga, Spain; 6 Endocrinology and Nutrition Department, Clinical University Hospital of Valladolid, 47003 Valladolid, Spain; dluisro@saludcastillayleon.es; 7 Investigation Centre Endocrinology and Nutrition, Faculty of Medicine, University of Valladolid, 47003 Valladolid, Spain; 8 Department of Endocrinology and Nutrition, Complejo Hospitalario Universitario de Ferrol (CHUF), 15405 Ferrol, Spain; diegobellido@gmail.com; 9 Department of Endocrinology and Nutrition, Virgen de la Victoria University Hospital, 29010 Malaga, Spain; 10 Department of Endocrinology and Nutrition, Quironsalud Málaga Hospital, 29009 Malaga, Spain

**Keywords:** ultrasound imaging, muscle composition, malnutrition, image segmentation, computer vision, machine learning, clinical diagnostics

## Abstract

**Background:** Malnutrition is a prevalent condition associated with adverse health outcomes, requiring the accurate assessment of muscle composition and fat distribution. **Methods:** This study presents a novel method for the automatic analysis of ultrasound images to estimate subcutaneous and visceral fat, as well as muscle, in patients with suspected malnutrition. The proposed system utilizes computer vision techniques to segment regions of interest (ROIs), calculate relevant variables, and store data for clinical evaluation. Unlike traditional segmentation methods that rely solely on thresholding or pre-defined masks, our method employs an iterative hierarchical approach to refine contour detection and improve localization accuracy. A dataset of abdominal and leg ultrasound images, captured in both longitudinal and transversal planes, was analyzed. **Results:** Results showed higher precision for longitudinal scans compared to transversal scans, particularly for length-related variables, with the Y-axis Vastus intermediate achieving a precision of 92.87%. However, area-based measurements demonstrated lower precision due to differences between manual adjustments by experts and automatic geometric approximations. **Conclusions:** These findings highlight the system’s potential for clinical use while emphasizing the need for further algorithmic refinements to improve precision in area calculations.

## 1. Introduction

Body composition is a fundamental aspect in determining an individual’s health status, influenced by environmental, genetic, and lifestyle factors [1]. Health professionals utilize body composition assessment as an essential tool to measure their patients’ nutritional status and to identify and diagnose conditions such as overweight, obesity, malnutrition, osteoporosis, and sarcopenic obesity [2].

From the perspective of diagnosing and treating nutrition-related diseases, the identification and measurement of fat, muscle, bone, and water are critical. Therefore, body composition evaluation is a crucial element, involving the separation of an individual’s total mass into fat mass and fat-free mass, where fat-free mass includes muscles, organs, ligaments, tendons, and water [3].

Ultrasound has established itself as a fundamental tool in clinical medicine due to its ability to evaluate body tissues and structures. This non-invasive, portable, cost-effective, real-time technique that does not emit ionizing radiation offers multiple advantages [4,5], although it presents the challenge of accurately interpreting the obtained data. Musculoskeletal ultrasound is a technique that uses high frequencies to obtain detailed images of skeletal muscle [6], allowing the observation of hierarchical relationships of soft tissues and their internal structures, as well as extracting characteristics such as muscle texture and echogenicity.

The analysis of these ultrasound images provides important clinical variables such as muscle thickness, the area of subcutaneous fat zones, and other variables directly related to a patient’s health status. These variables allow the precise evaluation of the evolution of certain pathologies. Through ultrasound, it is possible to obtain relevant information from variables such as thickness, echo intensity, echo variation, gray-level co-occurrence matrices, and echo texture parameters. These variables have been described in both healthy individuals and those with various diseases [7,8,9].

Accurately quantifying both muscle and fat tissue status is essential for assessing a patient’s health status. There is a growing interest in using ultrasound as a tool for body composition analysis, especially concerning sarcopenia [8]. Although several studies have suggested its potential, more research is still needed to validate its use in different populations [7,10]. Particularly, in malnourished patients, there is a shortage of studies evaluating body composition estimation using ultrasound images. Using ultrasound for body composition evaluation in this population could be promising, as it is a non-invasive and low-cost technique [11]. Identifying new biomarkers from ultrasound images could help in the early detection and monitoring of malnutrition and its complications, thereby improving the care and treatment of these patients [12].

The analysis of ultrasound images is a laborious process due to the presence of noise and the geometric complexity of the structures. Typically, this analysis requires a specialist to manually select the area of interest using interactive software, which can lead to errors, especially in patients with complex body structures. Moreover, this manual method is not suitable for analyzing large batches of serial images. For these reasons, an automated analysis method is necessary, allowing specialists to accurately obtain the variables of interest, thus improving diagnoses.

Various methods have been proposed to measure structural parameters through ultrasound images, especially muscle parameters. For instance, the MUSA method, proposed by Caresio and Salvi et al. [13], automates the measurement of muscle thickness in ultrasound images by detecting muscle fascicles using the Hough transform and an iterative heuristic search to identify aponeurosis contours and calculate muscle thickness. However, noise and the presence of blood vessels can hinder accuracy, and the method only measures muscle thickness. To address these limitations, TRAMA [14] was developed, using the Sobel operator and a Gaussian filter to segment cross-sectional ultrasound images of muscles, improving the identification of aponeuroses.

Zheng et al. proposed an automated method based on segmentation with Residual Shrinkage-Unet [15], achieving precise and comprehensive measurement of muscle parameters by eliminating complex noise. On the other hand, Burlina et al. [16] explored the automatic diagnosis of myositis using deep neural networks, achieving an accuracy of 79.2%. Marzola et al. [17] developed a method for diagnosing abnormal muscles based on the gray level of the cross-sectional area, achieving a classification accuracy of 91.5%. Zhou et al. [18] implemented a multitasking model, MMA-Net, for segmenting the cross-sectional muscle area and classifying abnormal muscles, achieving very high performance.

In general, the aforementioned automatic methods present several problems [15]; they are typically not fully automatic, relying on the manual selection of filters or areas of interest and are often highly sensitive to images with significant noise or the presence of unusual shapes.

In this article, we propose a method for the automatic analysis of ultrasound images based on computer vision. This system automatically segments areas of interest and analyzes the segmented images to automatically extract clinical variables of interest. This automated analysis method can reduce the diagnostic burden on physicians and provide an effective basis for subsequent treatment. The method is based on the use of computer vision and predefined ultrasound zone templates to automatically detect areas of interest.

## 2. Materials and Methods

In this article, we propose a method for the automatic analysis of ultrasound images aimed at estimating muscle composition in patients with suspected malnutrition using computer vision techniques.

### 2.1. Data

A large volume of anonymized images were compiled from two areas (abdomen and leg) and two planes (transverse and longitudinal). These images were captured using a commercially available portable ultrasound system with a 4–10 cm linear tube (UProbe L6C Ultrasound Scanner, Guangzhou Sonostar Technologies Co., Ltd., Guangzhou, China). From March to December 2022, consecutive patients aged 18 to 85 years admitted to the medical–surgical departments of the participating hospitals (excluding intensive care units [ICUs]) were eligible for inclusion if they were identified as being at risk of malnutrition during the first week of hospitalization and had provided informed consent.

All images were obtained from patients at collaborating hospitals [19,20], with ethical approval from the relevant committees. The study protocol was approved by the Ethics Committee for Clinical Research (CEIC) of the Health Council of the Andalusian Health Service (protocol code ALM-DRECO-2021-01, approval date 1 February 2022) and the individual Institutional Review Boards of the participating hospitals. Written informed consent was obtained from all patients.

#### Image Acquisition Procedure

The following procedure was followed for image acquisition: For the leg images, the patient lay down and the structure was located using the upper edge of the patella and the anterior superior iliac spine. The transducer was placed 14 cm above the patella, centered on the thigh. For transverse scans, the transducer was positioned perpendicular to the muscle fibers, and for longitudinal scans, it was rotated to align with the fibers once the tendon was located.

For the abdominal images, the patient also laid down and the midpoint between the xiphoid process and the navel was located. The transducer was placed transversely to the muscle fibers and then rotated to the longitudinal position.

### 2.2. Data Analysis and Processing

Once the images were obtained, the Open CV system proceeded with their analysis and processing. An Open CV system refers to a framework or software solution built using OpenCV (Open-Source Computer Vision Library), which provides tools and algorithms for real-time computer vision and image processing. The primary objective of this system is to segment images into specific regions, calculate the regions of interest (ROIs), and extract relevant features. Once extracted, the images are automatically stored in a database for subsequent consultation by clinical staff.

The images are uploaded to the system, allowing for the analysis of both individual images and batches. The system supports the inclusion of images in dcm, jpg, bmp, and png formats. The image processing involves the following stages.

#### 2.2.1. Image Segmentation

Using a predefined code in the image notation, specific areas for segmentation are identified. The image is divided into four categories: lateral leg, transverse leg, lateral abdomen, and transverse abdomen. This segmentation is crucial as it allows subsequent algorithm stages to focus on specific areas of the image, enhancing the accuracy and relevance of the analysis.

#### 2.2.2. Image Correction

Once segmented, the image undergoes an initial correction process. This process prepares the image for further analysis through color conversion and scale adjustments.

Conversion to Grayscale: The image is converted to grayscale. This conversion simplifies subsequent processing by reducing the amount of information to be handled, focusing solely on pixel intensity. In cases where the image format does not support direct grayscale conversion, the image is first converted to RGB before applying grayscale conversion using OpenCV functions. This ensures compatibility with various input formats while maintaining processing efficiency [21,22,23].

#### 2.2.3. Calculation of the Region of Interest (ROI)

The region of interest (ROI) is a specific section of an image containing relevant data for analysis. ROI calculation is performed using a threshold-based approach on horizontal and vertical axes.

Horizontal Thresholds: The algorithm begins by identifying the horizontal boundaries of the ROI. The process involves scanning the image from the center towards both the left and right sides to determine the limits where meaningful image data begins. The steps are as follows:

The midpoint of the image width is determined.

- A vertical range is selected to focus on the relevant anatomical structures.
- A backward scan (from the middle to the left) is performed to find the first column where pixel intensity exceeds a predefined threshold.
- A forward scan (from the middle to the right) is performed to find the last column where the pixel intensity is above the threshold.
- These two indices define the horizontal extent of the ROI.

Vertical Thresholds: Once the horizontal ROI is established, the algorithm determines the vertical boundaries using a similar thresholding approach:

- The midpoint of the image height is used as a reference.
- A downward scan is conducted to determine the lower boundary, which is the last row where significant pixel intensity is detected.
- An upward scan is performed to establish the upper boundary, where meaningful image data start.

The ROI is then extracted using these four coordinates.

#### 2.2.4. Image Filtering

The next step of the algorithm is filtering the image to remove noise and improve data quality. Noise filtering is applied after ROI detection rather than before to ensure that the noise removal process does not unintentionally alter key image features used for boundary detection. Since the ROI calculation relies on intensity differences within the original image, applying a filter too early could smooth out significant intensity transitions, leading to inaccurate ROI boundaries. By first determining the key anatomical regions and then reducing noise, the algorithm preserves essential structures while improving segmentation accuracy.

A bilateral filter is used to remove noise while preserving edges. A bilateral filter with a standard deviation for the range distance of 5 and a standard deviation for the color set at 0.1 is applied.

#### 2.2.5. Image Transformation

Finally, the filtered image is transformed to ensure consistent representation and facilitate subsequent calculations. The pixel values of the image are rescaled to an integer representation between 0 and 255. This step ensures that the image format is standardized for further processing, making it compatible with subsequent computational steps.

Template Matching and Contour Detection

After pixel value reescalation, template matching and contour detection are performed to identify and delineate the structures present in the image. This process ensures accurate segmentation by leveraging predefined templates corresponding to specific anatomical structures.

For contour detection, the binarized image of the ROI is used. This process utilizes the open-source package OpenCV version 4.8.074 in Python 3.10.

The core of the detection process involves searching for predefined templates across different regions within the image. The method used is TM_CCORR, which performs correlation-based matching between the input image and template images. The image is processed iteratively, where multiple versions of a template are tested until a match is found. If a template does not provide a valid match, a fallback mechanism is triggered to explore alternate templates with slight variations in scale or contrast, ensuring that a match is eventually identified.

The algorithm processes the image in both directions, from the deeper anatomical structures towards the surface (bottom–up) and from the surface to deeper structures (top–down). This bidirectional approach increases the chances of accurate detection by ensuring that no structure is overlooked due to image variability or noise.

When a match is found, the algorithm extracts the bounding box coordinates (top-left and bottom-right points) and overlays them onto the image for visualization. If no match is initially found, alternative variations in the template are tested until a suitable match is identified.

Once all relevant structures are detected, the contours of the identified regions are compared against the predefined template boundaries. To avoid false positives, constraint-based validation is applied to ensure that detected structures align correctly with expected anatomical positions. The contours undergo a final refinement process using additional morphological operations to smooth edges and remove outliers. The extracted contours are then stored for further analysis or visualization, guaranteeing consistency across different scans.

**Figure 1:**
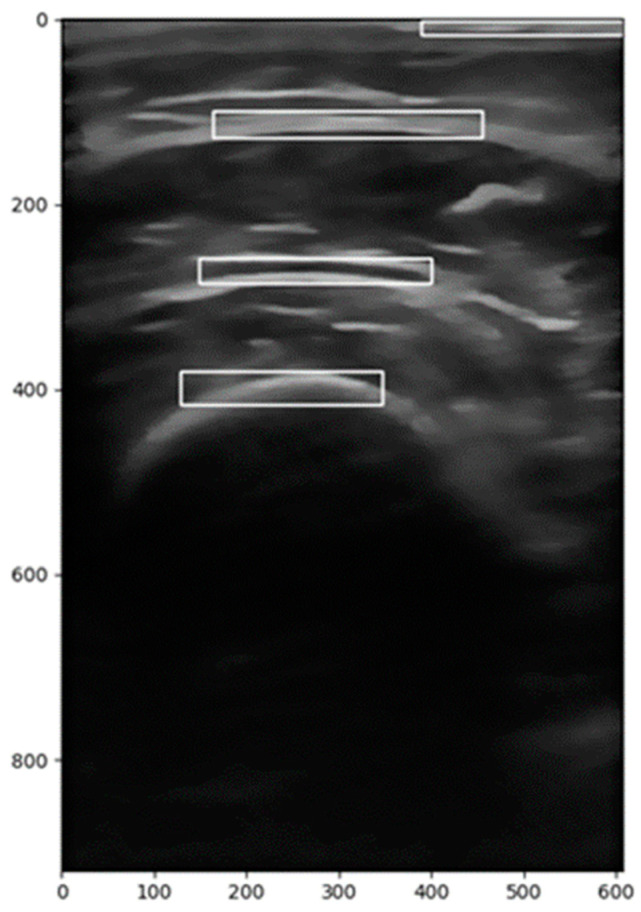
Identified anatomical regions using predefined segmentation templates of ROI (range of interest) in ultrasound images.

This approach allows for the precise identification and delineation of the structures within each region of interest while ensuring consistency across both search directions. The following Figure 1 shows the delimited zones detected using the templates.

#### 2.2.6. Calculation of Distances and Areas

With the positions of the detected contours, the algorithm proceeds to calculate the relevant distances and areas. Distances between different zones of interest, such as the distance between muscles and skin, are calculated. This involves calculating the distance between the edges of the contours to determine the height. The width is the width of the image, which is previously defined during the initial processing.

Once the height and width of the zone are determined, the area is calculated using rectangular and elliptical approximations, depending on the shape of the zone. For elliptical areas, the direct least-squares method [20] is used to fit the ellipse shape to the image shape.

#### 2.2.7. Feature Extraction

Finally, the algorithm extracts various variables of interest from the obtained data and the initial image

### 2.3. Variables of Interest

In this article, various ultrasound variables were extracted and classified into two main categories: length and area variables and image characteristic variables.

#### 2.3.1. Length and Area Variables

This study included four types of images: transverse abdomen, longitudinal abdomen, transverse leg, and longitudinal leg images. Length and area variables allowed for the quantification of physical dimensions within the images. Table 1 includes the variables depending on the area and cut.

#### 2.3.2. Image Characteristic Variables

In addition to the length and area variables, various image characteristic variables were analyzed for all the mentioned types of images. These variables were based on the textural and statistical properties of the ultrasound images:

- Angular Second Moment (ASM): Also known as uniformity or energy, this measure evaluates the repetition and uniformity of gray levels. A high ASM value indicates greater uniformity.
- Contrast: This measures the variation in gray levels between neighboring pixels. High contrast indicates a clear distinction between light and dark areas.
- Correlation: This assesses the linear dependence between the gray levels of pixels in a specific direction. A value of 0 indicates no correlation.
- Dissimilarity: This measures the difference between the gray levels of adjacent pixels. A high value reflects greater variability.
- Entropy: This is used to evaluate the lack of uniformity in the image texture. Higher entropy indicates lower uniformity.
- Histogram: This represents the normalized distribution of gray levels in the image, providing the mean of these levels.
- Homogeneity: This indicates the similarity between the gray levels of the pixels in the image, being a measure of textural uniformity.

### 2.4. Data Storage

The calculated values of the characteristics were returned and stored in a database for further analysis and use in research or medical diagnostics.

#### Interface

Once data are in the database, the end user can query and download all extracted variables through a web interface designed for this purpose. Users can query individual patients or groups based on hospitals, visits, regions, cuts, etc.

### 2.5. Statistical Methods

The Bland–Altman analysis method was employed to evaluate the agreement between two measurement techniques. This approach used a scatter plot to display the differences between the two methods against their mean, offering a clear view of the agreement, bias, and limits of agreement. Statistical analysis and calculations were conducted using RStudio 2023.06.0 Build 421 (Posit Software, PBC, formerly RStudio, PBC; the open-source data science company, located at 250 Northern Ave, Suite 420, Boston, MA, USA 02210, 844-448-1212).

## 3. Results

The analysis of ultrasound muscle segmentation for subcutaneous and visceral fat, as well as muscle characteristics, yielded the following results. Table 2 (CI 95%) summarizes the adjusted precision percentages for each evaluated variable. The variables were compared through manual analysis performed by an expert operator using a modified version of the application that allowed for area selection.

For the abdominal region, subcutaneous fat measurements showed higher precision in longitudinal scans compared to transversal ones, with the total subcutaneous fat reaching 71.42% and superficial subcutaneous fat at 68.77%. Precision for peritoneal fat was slightly lower, reaching 63.00%. Transversal cuts in the abdominal region demonstrated consistently lower precision, particularly for superficial subcutaneous fat, which reached 55.65%.

In the leg region, both longitudinal and transversal scans demonstrated variability in precision. For longitudinal cuts, the Y-axis Rectus femoris achieved a precision of 80.43%, while the precision for the Rectus femoris area was significantly lower at 43.29%. Similarly, for transversal cuts, the intermediate area of the Vastus reached only 41.05%, while the Y-axis Vastus intermediate exceeded expectations with a precision of 92.87%.

For the calculation of precision, the deviation percentage between the automated measurements and the manually annotated reference values was used. The formula accounted for the mean absolute percentage error, allowing for a quantitative comparison of precision across different structures.

Overall, area analyses showed lower precision compared to manual calculations. However, areas such as the Rectus femoris area and subcutaneous fat superficial demonstrated room for improvement, suggesting potential algorithmic refinement or dataset adjustments.

**Figure 2:**
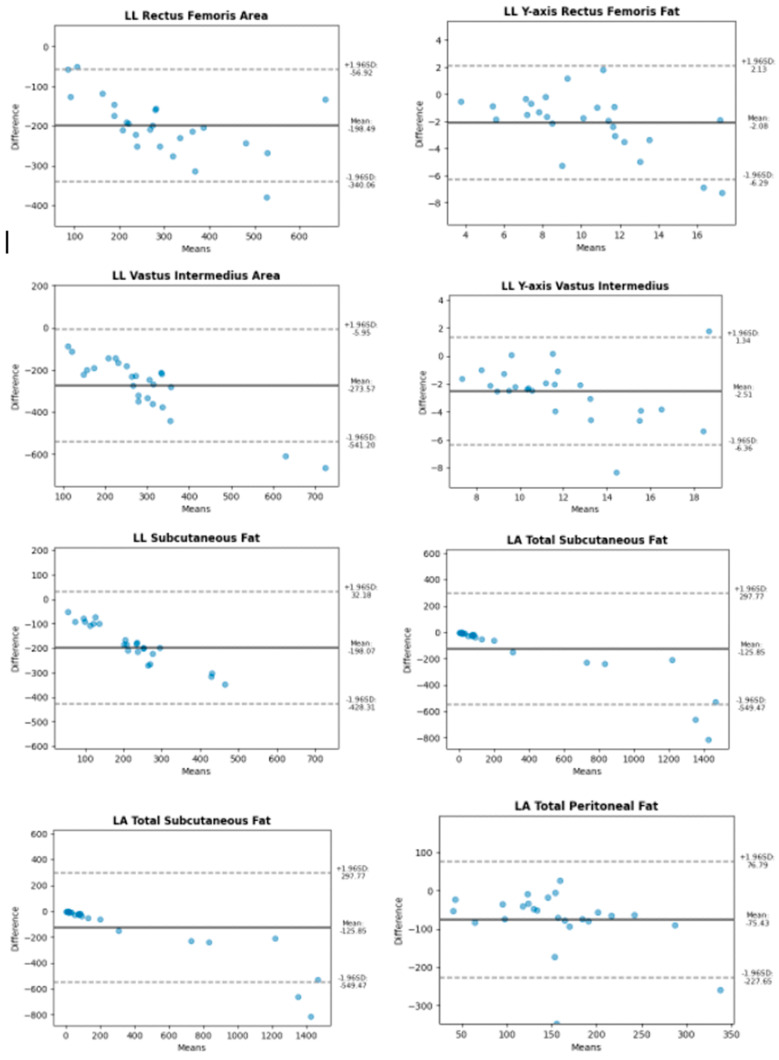
Bland–Altman plots for longitudinal scans of muscle and subcutaneous and visceral fat tissues.

**Figure 3:**
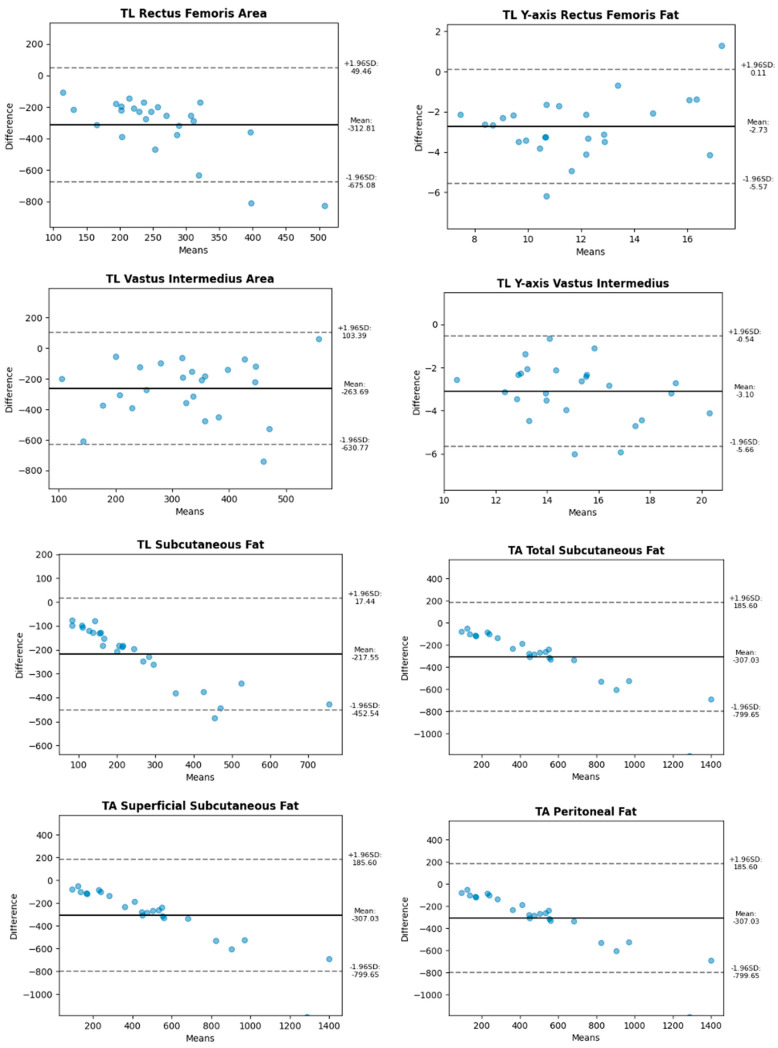
Bland–Altman plots for transverse (axial) scans of muscle, subcutaneous fat, and visceral fat tissues.

Bland–Altman plots were used to compare these measurements and visually assess agreement. Specifically, Figure 2 and Figure 3 illustrate the agreement between two measurement techniques, where Figure 2 corresponds to longitudinal scans for both the leg and abdominal regions, while Figure 3 represents the transverse cuts for the same anatomical areas.

## 4. Discussion

The proposed method for the automatic analysis of ultrasound images successfully segmented muscle structures and estimated fat composition in patients with suspected malnutrition. The results demonstrate that longitudinal scans generally provided higher precision compared to transversal scans, particularly in measuring subcutaneous fat and muscle axes. For example, the Y-axis Vastus intermediate in the leg region achieved the highest precision of 92.87%, while other variables such as Rectus femoris area and subcutaneous fat superficial showed significant room for improvement.

One key observation is the consistent discrepancy in precision for area calculations when compared to manual analysis performed by expert operators. This can be attributed to the differences in measurement approaches: manual analysis allows the precise adjustment of areas, while the automatic method approximates the shapes to geometric figures, such as rectangles or ellipses. These approximations inherently introduce variability and limit the precision of the algorithm in delineating complex anatomical regions. This study underscores the importance of incorporating expert-annotated datasets and exploring hybrid approaches that combine manual and automated analysis to bridge the gap in precision and ensure the method’s clinical applicability. The system demonstrates consistent performance across various imaging devices, ensuring robustness in different clinical settings.

However, several factors can introduce errors in the segmentation process, which must be considered when interpreting the results. One primary source of error is the variability in fat–muscle boundaries, as differences in tissue echogenicity and contrast impact the effectiveness of template matching. Patient-specific anatomical variations, including differences in muscle thickness and fat distribution, can lead to inconsistencies in contour detection.

Despite these limitations, the system demonstrated its potential as a reliable tool for estimating relevant variables in ultrasound images, particularly for length-related measures. First, it was used exclusively with patients with malnutrition associated with disease, which could initially suggest a limitation in its generalizability. However, the model was designed with a template-matching approach that allowed adaptation to various anatomical variations. As a result, while it was optimized for malnourished individuals, its effectiveness was also observed in patients with different BMI ranges, ensuring broader applicability in clinical settings. Second, while ultrasound (US) is not widely utilized for assessing muscle mass in patients with disease-related malnutrition (DRM), clinical guidelines recommend its use, and a recent study demonstrated good accuracy of this automatic method based on IA analysis [24]. Third, this study focused on a single muscle, the rectus femoris. Lastly, the automated method used requires replication and cross-validation with another cohort and comparison against MRI/CT. Despite these limitations, this study has notable strengths: the ultrasound imaging of the RF was meticulously standardized.

Future work should focus on refining the segmentation and shape-fitting algorithms to reduce discrepancies in area calculations. Also, in future work, a power analysis should be conducted to determine the appropriate number of experts required for a robust comparison. Additionally, increasing the diversity and quality of the training dataset could enhance the system’s robustness across different patient profiles and ultrasound devices. As an alternative to the current computer vision-based approach, future research should also explore deep learning-based segmentation techniques. A neural network model based on U-Net is being considered for future development, as it has shown promising results in medical image segmentation.

## 5. Conclusions

In this report, an automated method for the analysis of ultrasound images was developed to estimate muscle composition in patients with suspected malnutrition. The approach leverages computer vision techniques to automatically segment regions of interest and extract clinically relevant variables, reducing manual workload and improving diagnostic accuracy. The system processes images through stages of segmentation, correction, filtering, normalization, and contour detection, utilizing OpenCV and bilateral filtering. Key variables, including thickness and texture features, are extracted and stored in a database for clinical use. Despite challenges with noisy or low-quality images, the method shows promise for enhancing the assessment of body composition, particularly in malnourished patients. Future research will focus on improving robustness by use of advanced neural networks and the automatic detection of the area and type of scan to eliminate the need for file annotation.

## Figures and Tables

**Table 1:** Length and area variables according to cut and zone.

	Abdomen	Leg
Longitudinal	Total Subcutaneous Fat Superficial Subcutaneous Fat Peritoneal Fat	Y-axis Anterior Rectus X-axis Anterior Rectus Anterior Rectus Area Y-axis Vastus Intermedius X-axis Vastus Intermedius Vastus Intermedius Area
Transverse	Total Subcutaneous Fat Superficial Subcutaneous Fat Peritoneal Fat	Y-axis Anterior Rectus X-axis Anterior Rectus Anterior Rectus Area Y-axis Vastus Intermedius X-axis Vastus Intermedius Vastus Intermedius Area

**Table 2:** Accuracy of the analysis.

Member	Section	Type	Accuracy
Abdomen	Longitudinal	Subcutaneous fat, total	71.42
		Subcutaneous fat, superficial	68.77
		Peritoneal fat	63.0
	Transversal	Subcutaneous fat, total	54.3
		Subcutaneous fat, superficial	55.65
		Peritoneal fat	51.02
Leg	Transversal	Y-axis Rectus femoris	78.73
		Rectus femoris area	28.09
		Y-axis Vastus intermediate	81.65
		Vastus intermediate area	43.31
	Longitudinal	Subcutaneous fat	40.82
		Y-axis Rectus femoris	83.85
		Rectus femoris area	46.33
		Y-axis Vastus intermediate	81.39
		Vastus intermediate area	36.52

## Data Availability

Data are unavailable due to privacy and ethical restrictions.
